# Chemical Characterization, Antitumor, and Immune-Enhancing Activities of Polysaccharide from *Sargassum pallidum*

**DOI:** 10.3390/molecules26247559

**Published:** 2021-12-13

**Authors:** Yi Gao, Yizhen Li, Yunze Niu, Hao Ju, Ran Chen, Bin Li, Xiyun Song, Lin Song

**Affiliations:** 1 College of Marine Science and Engineering, Qingdao Agricultural University, Qingdao 266109, China; gaoyi@qau.edu.cn (Y.G.); qdaulb@163.com (B.L.); 2 College of Life Sciences, Qingdao Agricultural University, Qingdao 266109, China; lyzliyizhen@163.com (Y.L.); niuyunze1997@163.com (Y.N.); jh19981110@163.com (H.J.); cr1164365956@163.com (R.C.); 3 College of Agronomy, Qingdao Agricultural University, Qingdao 266109, China; songxy@qau.edu.cn; 4 College of Marine Science and Biological Engineering, Qingdao University of Science and Technology, Qingdao 266042, China; 5 Shandong Provincial Key Laboratory of Biochemical Engineering, Qingdao 266042, China

**Keywords:** seaweed, *Sargassum pallidum*, polysaccharides, antitumor activity, immune-enhancing activity

## Abstract

Searching for natural products with antitumor and immune-enhancing activities is an important aspect of cancer research. *Sargassum pallidum* is an edible brown alga that has been used in Chinese traditional medicine for the treatment of tumors. However, the purification and application of its active components are still insufficient. In the present study, the polysaccharides from *S. pallidum* (SPPs) with antitumor and immune-enhancing activities were isolated and purified, and five polysaccharide fractions (SPP-0.3, SPP-0.5, SPP-0.7, SPP-1, and SPP-2) were obtained. The ratio of total saccharides, monosaccharide composition, and sulfated contents was determined, and their structures were analyzed by Fourier transform infrared spectroscopy. Moreover, bioactivity analysis showed that all five fractions had significant antitumor activity against three types of cancer cells (A549, HepG2, and B16), and can induce cancer cell apoptosis. In addition, the results indicated that SPPs can enhance the proliferation of immune cells and improve the expression levels of serum cytokines (IL-6, IL-1β, iNOS, and TNF-α). SPP-0.7 was identified as the most active fraction and selected for further purification, and its physicochemical properties and antitumor mechanism were further analyzed. Transcriptome sequencing result showed that SPP-0.7 can significantly induce the cell apoptosis, cytokine secretion, and cellular stress response process, and inhibit the normal physiological processes of cancer cells. Overall, SPPs and SPP-0.7 may be suitable for use as potential candidate agents for cancer therapy.

## 1. Introduction

Cancer is one of the most serious diseases that threaten human health and life, and is characterized by the growth of malignant tumors [1,2]. Currently, the most common treatment of cancer is a combination of chemotherapy, radiotherapy, and surgical intervention [3]. However, these treatments inevitably damage the body’s normal immune system and often cause serious side effects, such as nausea, vomiting, fatigue, anemia, and anorexia [4,5]. The damage of the immune system can easily lead to tumor recurrence, ultimately leading to the failure of cancer therapy [3]. Immunotherapy to improve immunity has become a clinical treatment approach for cancer [6,7]. A competent immune system can recognize and damage fast-growing tumor cells and reduce the probability of side effects [8]. Therefore, research has focused on the development of new treatment strategies and identification of natural products with antitumor and immune-enhancing properties to replace existing drugs.

Polysaccharides are composed of monosaccharide units connected by glycosidic bonds. They are considered to be important biological response modifiers with good therapeutic and health promotion effects [9]. It has been shown that they play important roles in organisms and are widely involved in cellular immune defense, differentiation, adhesion, and proliferation [10,11]. As immunomodulators, polysaccharides can directly or indirectly interact with immune cells, triggering various cellular or molecular events, leading to immunoregulatory effects [12,13]. Correspondingly, tumor cells, pathogens, and other harmful factors secreted by tumor cells are killed or neutralized by the immune system [13]. Several polysaccharides, such as lentinan, astragalus polysaccharide, ganoderma atrum polysaccharide, and anoderma lucidum polysaccharide, have been used as immunotherapeutics for cancer therapy [14,15].

*Sargassum pallidum* (Turn.) C. Agardh is an edible brown alga that grows along the coasts of China, Korea, and Japan [16]. To date, this seaweed has been widely used to treat tumors in Chinese traditional medicine for thousands of years [17]. The ancient Chinese herbal book “Herbal Classic of Shen Nong” recorded that “Haizao” (*S. pallidum* and *Hizikia* *fusiforme*) can treat thyroid-related tumors, relieve turbid phlegm and blood stasis, and clear interior heat [18]. During the Ming Dynasty, the medical book “Compendium of Materia Medica” recorded that *S. pallidum* can heal solid tumors, reduce swelling and clear interior heat [18]. The “Haizao Yuhu decoction” (HYD) is a formula that has been used in Chinese traditional medicine for approximately 500 years and has been reported to efficiently treat thyroid-related diseases, including thyrophyma [19]. The “Haizao” (seaweed) in HYD refers to *S. pallidum*. Modern pharmacological studies have shown that polysaccharides extracted from *S. pallidum* have a wide range of beneficial health-promoting properties, such as antioxidant, hypolipidemic, antibacterial, and antitumor activities [20,21,22,23]. However, the components of *S. pallidum* polysaccharides need to be further purified, especially those in the fractions with antitumor and immune-enhancing activities, which are expected to be used as immunotherapeutic drugs in cancer therapy in the future.

The aim of this study was to purify polysaccharides and identify natural compounds with antitumor and immune-enhancing properties. By comparing the antitumor effect, defined as the inhibition of the growth rate of three types of cancer cells, and the immune-enhancement effect, defined as the promotion of proliferation of mouse macrophages (RAW264.7) and lymphocytes, the polysaccharide fraction with the best activity was selected for further purification. Additionally, the mechanisms underlying its antitumor and immune-enhancement activities were investigated. The results will provide valuable information for future studies of *S. pallidum* polysaccharides (SPPs) as immunomodulators and potential compounds for tumor therapy.

## 2. Results

### 2.1. Purification and Chemical Characteristics of S. pallidum Polysaccharides 

Crude polysaccharides were obtained with a yield of 6.74% (*w*/*w*) from *S. pallidum* through hot-water extraction, ethanol precipitation, dialysis, and lyophilization. The crude polysaccharides were further deproteinized and chromatographed using DEAE-Sepharose fast-flow column (GE Healthcare, New York, NY, USA). Five fractions were obtained, namely SPP-0.3, SPP-0.5, SPP-0.7, SPP-1, and SPP-2, with yields of 11.9%, 7.5%, 5.9%, 9.2%, and 2.3%, respectively. The ratios of total saccharides in the five fractions were 43.2%, 67.16%, 73.27%, 62.59%, and 63.45%, respectively. Sulfate contents in SPP-2 (13.79%) and SPP-0.7 (9.57%) were higher than those in SPP-0.3 (5.39%), SPP-0.5 (6.88%), and SPP-0.3 (8.80%) (Table 1). 

The molar ratios of the monosaccharides in the polysaccharide fractions were further characterized. The most abundant component in SPP-0.3, SPP-1, and SPP-2 was rhamnose, whereas in SPP-0.5 and SPP-0.7, fucose was the most abundant component. Individual variations with different ratios of monosaccharides were found (Table 1), which indicated that the five polysaccharide fractions had different chemical compositions.

### 2.2. Fourier Transform Infrared (FT-IR) Spectrum Characterization

**Figure 1:**
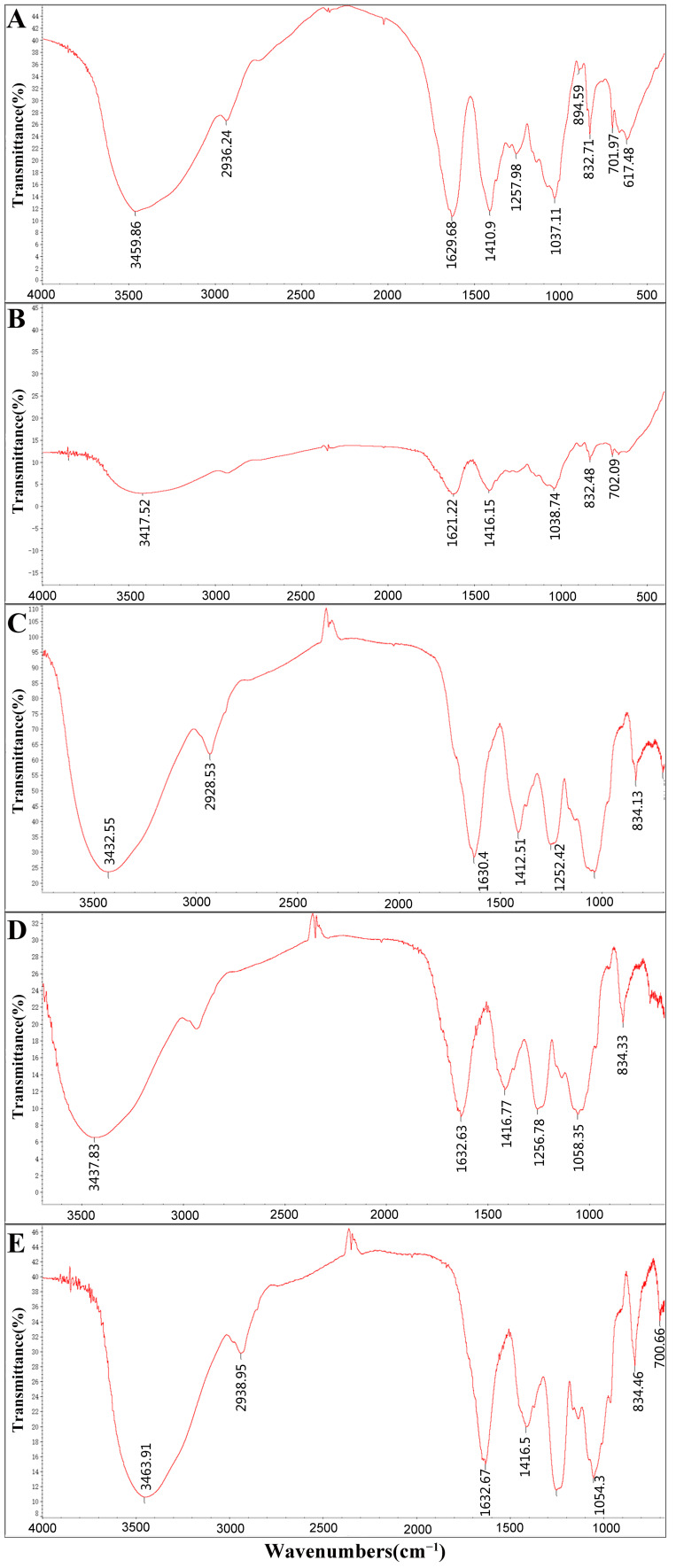
FT-IR spectra of five polysaccharide fractions extracted from *S. pallidum*. SPP-0.3 (**A**), SPP-0.5 (**B**), SPP-0.7 (**C**), SPP-1 (**D**), and SPP-2 (**E**).

The FT-IR spectra revealed that all five fractions shared several absorption peaks (Figure 1), which were considered to correspond to the α-type glycosidic linkages (830 cm^−1^), C–O–H deformation vibrations (1037–1054 cm^−1^), water absorption bands at 1623–1632 cm^−1^, stretching vibrations of –COO– (1410 cm^−1^), C–H stretching vibrations (2930 cm^−1^), and O-H stretching vibrations (3417–3459 cm^−1^) [24,25,26]. In addition, there were some differences in the FT-IR spectra of the five fractions; for example, the S=O asymmetric stretching vibrations at 1250 cm^−1^ were absent in SPP-0.5 [27].

### 2.3. Antitumor Activity of Five Polysaccharide Fractions 

**Figure 2:**
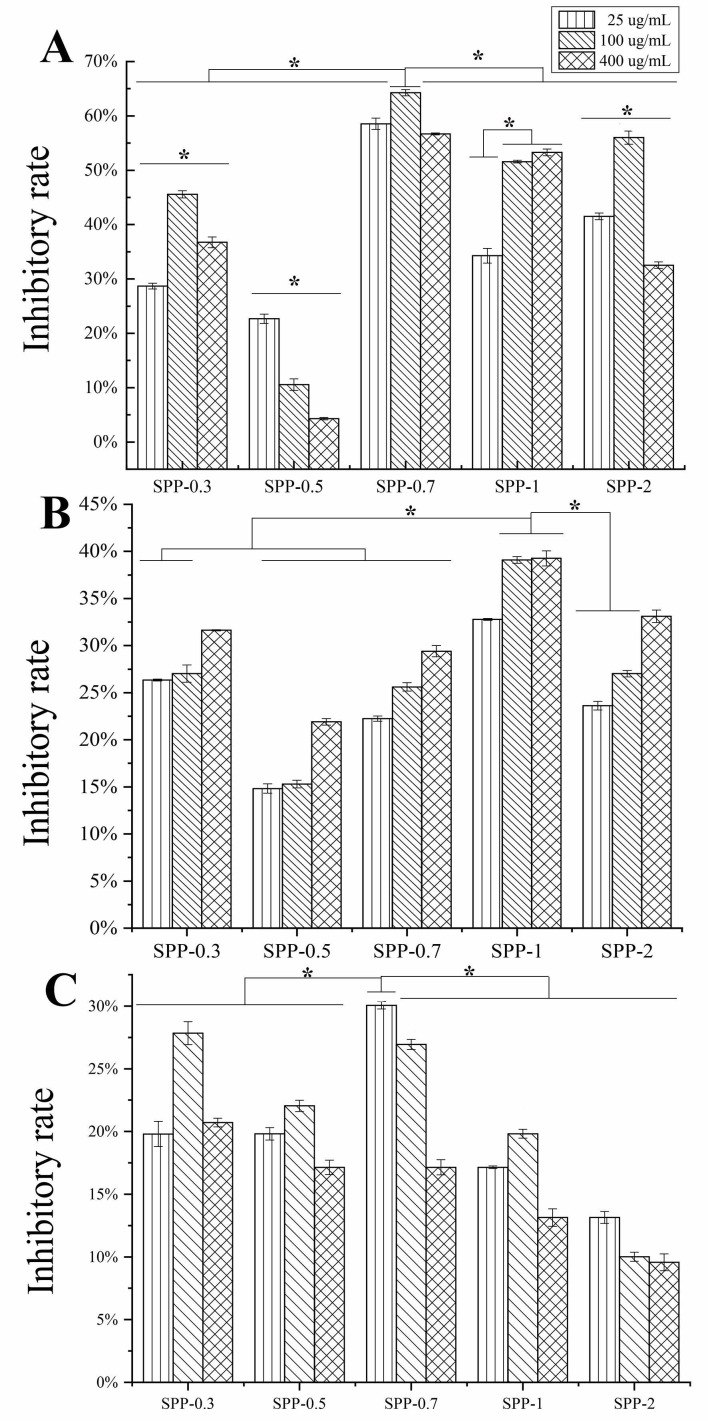
Inhibitory effects of five polysaccharide fractions with different concentrations (25, 100, and 400 μg/mL) on proliferation of A549 (**A**), HepG2 (**B**) and B16 (**C**) cells (mean ± SD). The asterisk (*) on the bars indicated the significance difference (*p* < 0.05) between the inhibitory rate of each group according to one-way ANOVA test.

The antitumor activity of different concentrations (25, 100, and 400 μg/mL) of the five polysaccharide fractions on the growth of three different cell lines, A549, HepG2, and B16, was evaluated. Exposure to SPPs resulted in decreased proliferation in all cell lines (Figure 2). For A549 cells, the inhibitory effects of the different fractions were in the order of SPP-0.7 > SPP-1 > SPP-2 > SPP-0.3 > SPP-0.5. At 100 μg/mL, SPP-0.7 exhibited the highest growth-inhibitory effect against A549 cells with an inhibitory rate of 64.28 ± 0.57%, which was significantly higher than that of the other fractions (Figure 2A). The order of the inhibitory rates of the different fractions on HepG-2 cells was SPP-1 > SPP-0.3 > SPP-2 > SPP-0.7 > SPP-0.5. The inhibition rate of SPP-0.7 on HepG2 cells increased slightly with the increase in concentration, and SPP-1 exhibited the highest growth-inhibitory effects against HepG-2 cells, with an inhibitory rate of 39.26 ± 1.82% at 400 μg/mL (Figure 2B). For B16 cells, the inhibitory effects of different fractions were in the order of SPP-0.7 > SPP-0.5 > SPP-1 > SPP-2 > SPP-0.5; SPP-0.7 had the highest antitumor activity (30.06 ± 0.29%) against B16 cells at 25 μg/mL (Figure 2C). The results indicated that the polysaccharide fractions SPP-0.7 and SPP-1 exhibited the highest specific antitumor activity.

### 2.4. Immunologic Modulation by the Five Polysaccharide Fractions

#### 2.4.1. The Effect of the Five Polysaccharide Fractions on Macrophage (RAW264.7) Proliferation

**Figure 3:**
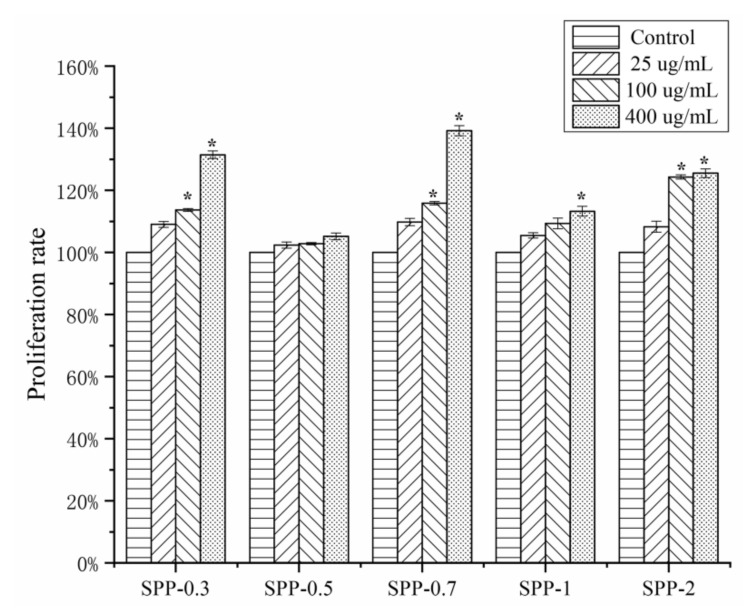
Effects of five polysaccharide fractions with different concentrations (25, 100, and 400 μg/mL) on proliferation of macrophage (RAW264.7) cells (mean ± SD). The asterisk (*) on the bars indicated the significance difference (*p * <  0.05) in proliferation rate between experimental group and control group according to one-way ANOVA test.

RAW264.7 cells are a good model to study polysaccharide immunomodulatory activity. As shown in Figure 3, the five polysaccharide fractions were not cytotoxic to RAW264.7 macrophages but enhanced their proliferation at concentrations ranging from 25 to 400 μg/mL in a concentration-dependent manner (*p* < 0.05), except for SPP-0.5. At a concentration of 400 μg/mL, the proliferation rates of cells treated with SPP-0.3, SPP-0.5, SPP-0.7, SPP-1, and SPP-2 were 131 ± 1.21%, 105 ± 1.09%, 139 ± 1.61%, 113 ± 1.58%, and 125 ± 1.39%, respectively. The polysaccharide fraction SPP-0.7 exhibited the highest macrophage proliferation activity.

#### 2.4.2. The Effect of the Five Polysaccharide Fractions on Lymphocyte Proliferation

**Figure 4:**
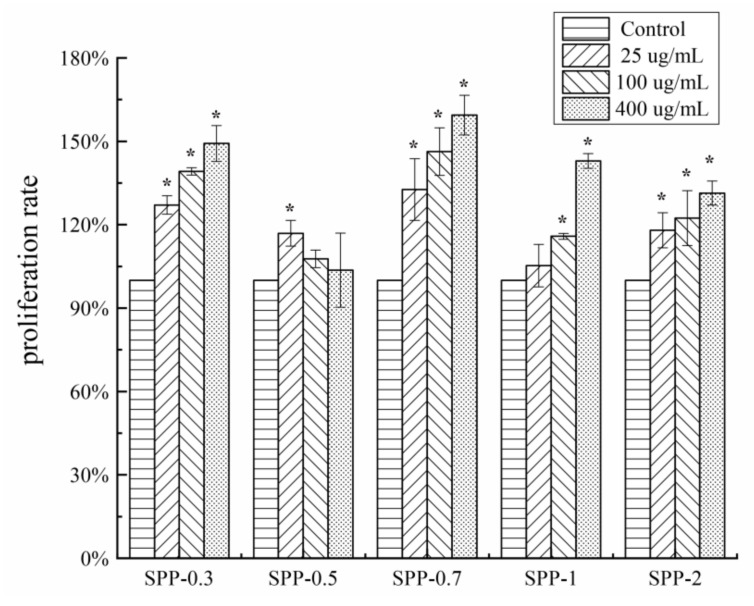
Mouse spleen cell proliferation effects of five polysaccharide fractions with different concentrations (25, 100, and 400 μg/mL) (mean ± SD). The asterisk (*) on the bars indicated the significance difference (*p * <  0.05) in proliferation rate between experimental group and control group according to one-way ANOVA test.

Lymphocyte proliferation is another indicator of immune activation. Therefore, the response of lymphocytes to the treatment with the polysaccharide fractions was examined. As shown in Figure 4, SPP (25, 100, and 400 μg/mL) treatment significantly promoted the proliferation of lymphocytes in a concentration-dependent manner, except for SPP-0.5, suggesting that SPPs significantly stimulate lymphocyte proliferation. Furthermore, 400 μg/mL SPP-0.7 had the strongest stimulatory effect, with a cell proliferation level of 159.43 ± 7.14%.

### 2.5. Purification and Nuclear Magnetic Resonance (NMR) Analysis of SPP-0.7

**Figure 5:**
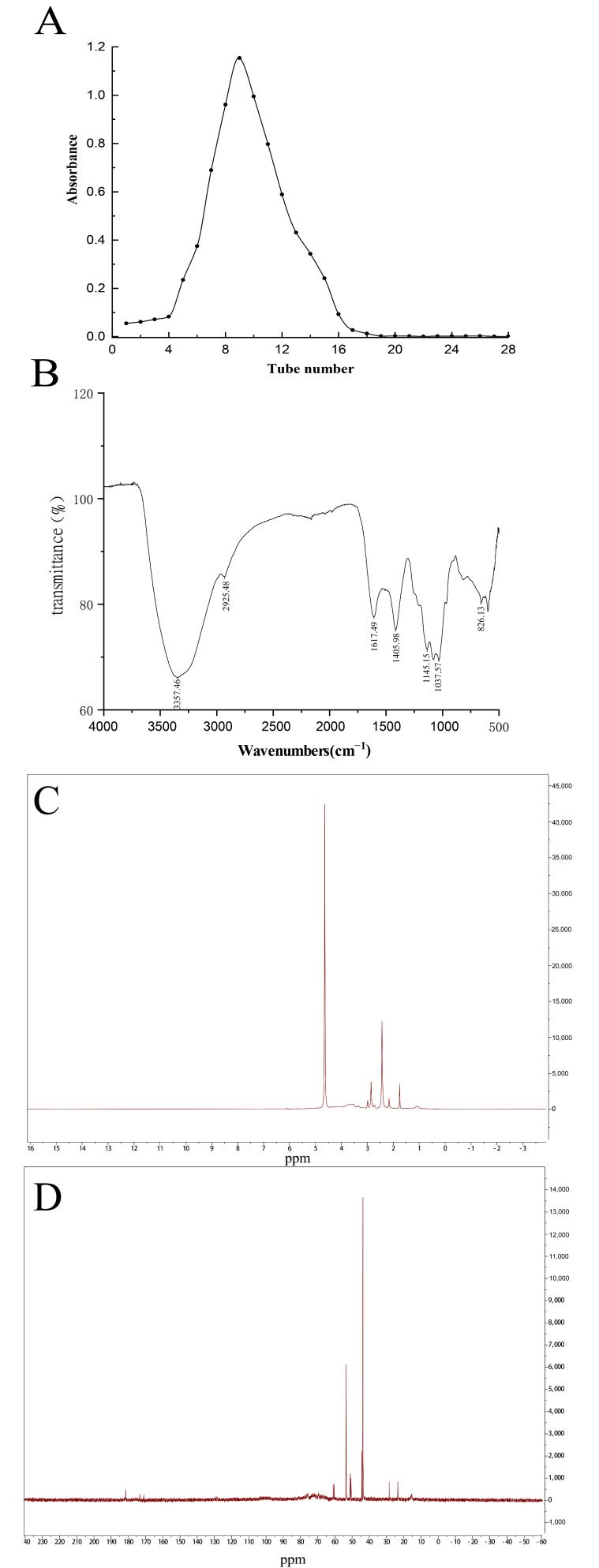
Purification and NMR spectra of SPP-0.7. (**A**) The elution curve of SPP-0.7 on the Sephadex G-100 gel chromatography column, (**B**) FT-IR spectra of SPP-0.7A, (**C**) ^1^H-NMR spectrum of SPP-0.7A, and (**D**) ^13^C-NMR spectrum of SPP-0.7A.

Based on the previous biological assays, fraction SPP-0.7 had the strongest antitumor and immune-enhancing effect. SPP-0.7 was further purified using a Sephadex G-100 gel filtration column (Solarbio, Beijing, China) according to the molecular distribution. The purified fraction was concentrated, dialyzed, freeze-dried, and named SPP-0.7A. The yield of SPP-0.7A was 41.1%. The ratio of total saccharides in SPP-0.7A was 71.56 %, the sulfated contents was 10.11 %, and monosaccharide composition was Man, 1.015; Gal, 0.289; Fuc,1. A single symmetrical peak was detected in the elution curve (Figure 5A), indicating that SPP-0.7A is a homogeneous polysaccharide.

The structural characteristics of SPP-0.7A were further analyzed by FT-IR (Figure 5B) and NMR spectroscopy (Figure 5C,D). The results showed that the purified SPP-0.7A has similar physical and chemical properties to SPP-0.7. The chemical shift at 5.2 ppm in ^1^H NMR spectrum of SPP-0.7A corresponded to the galactose signal [28] (Figure 5C). The signal of alkane region was present at 1.1 ppm weak signal [29]. The signal at 1.5–3 ppm represented the carbonyl region [30]. The interference peak signal of deuterated solvent existed at 4.7 ppm.

The signals from -22 to 55 ppm in the ^13^C-NMR spectrum corresponded to the sp^3^ hybrid carbon (Figure 5D). Among them, the signal of fucose C6 was present at 16.05 ppm [31]. Signals at 40–60 ppm were considered to represent C–OCCH_3_ [30]. There was a signal at 61 ppm that belonged to the unsubstituted C6 [31]. Signals from 65 to 80 ppm were due to C2–C5 [32]. The signals between 72 and 80 ppm were attributed to the characteristic of the pyran ring, which indicated that the monosaccharides in SPP-0.7A exist in the form of pyran rings [33]. There were three weak signals at 170 to 185 ppm, which correspond to carbonyl C(C=O) [34].

### 2.6. The Mechanism of the Antitumor and Immune-Enhancing Activities of SPP-0.7A

#### 2.6.1. Apoptosis Analysis

**Figure 6:**
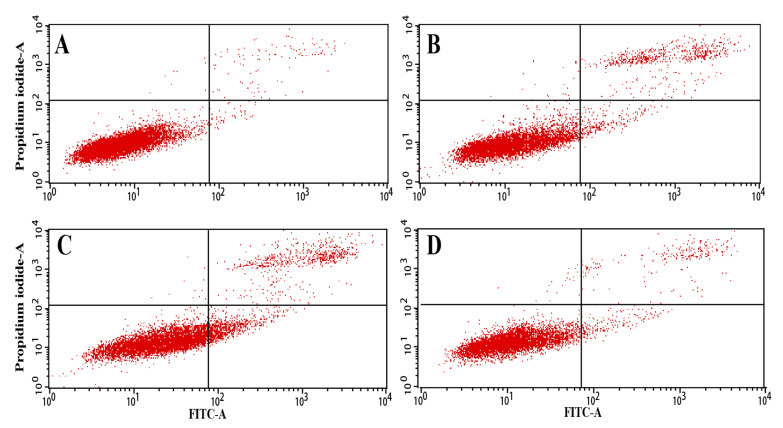
Effect of SPP-0.7A on A549 cell apoptosis, assessed by flow cytometry analysis. Control group (**A**), 25 μg/mL (**B**), 100 μg/mL (**C**), and 400 μg/mL (**D**).

To assess whether the growth inhibitory effects of SPPs on cancer cells were associated with apoptosis, the most active fraction SPP-0.7A was selected to measure the apoptosis-inducing effect on cancer cells (Figure 6). After 48 h of incubation with different concentrations of SPP-0.7, the apoptosis rates of A549 cells in the low-concentration (25 μg/mL), medium-concentration (100 μg/mL), and high-concentration (400 μg/mL) groups were 7.99%, 8.01% and 3.62%, respectively, which were significantly higher than the apoptosis rate in the control group (1.12%). These data suggested that SPP-0.7 remarkably induces the apoptosis of cancer cells, which was consistent with the results of the cancer cell proliferation assay.

#### 2.6.2. Cytokine Expression Analysis

**Figure 7:**
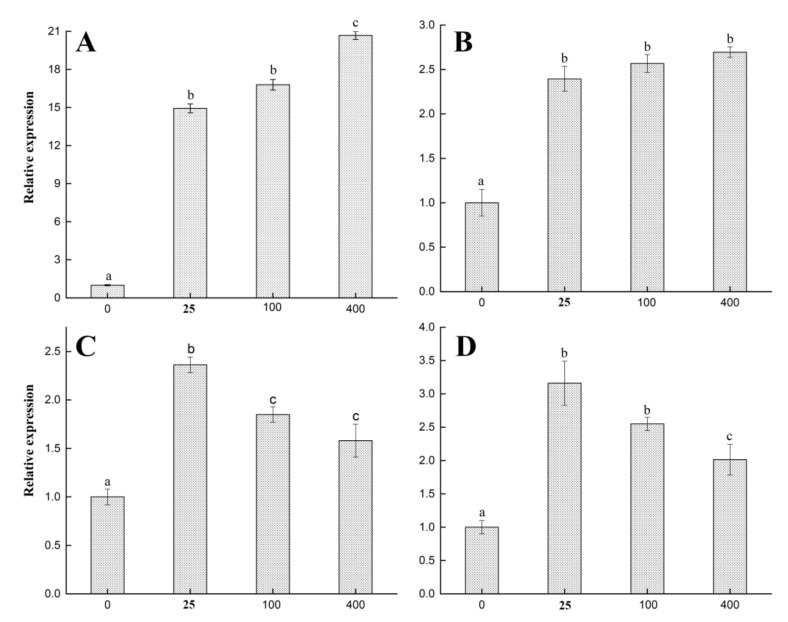
Effects of SPP-0.7A on expression level of serum cytokines, IL-6 (**A**), IL-1β (**B**), TNF-α (**C**), and iNOS (**D**) in RAW 264.7 cells. All data are expressed as mean ± SD. Significant difference (*p* < 0.05) between groups according to one-way ANOVA test are labeled with different letters.

To study the underlying mechanism of the immune-enhancing activity of SPP-0.7A, the expression levels of serum cytokines (interleukin-6 [IL-6], interleukin-1 beta [IL-1β], inducible nitric oxide synthase [iNOS], and tumor necrosis factor-α [TNF-α]) were detected by real-time fluorescent quantitative PCR (RT-PCR). SPP-0.7A treatment significantly increased the expression of all cytokines (Figure 7). For IL-6 and IL-1β, there was a concentration-dependent increase in the expression levels. At 400 μg/mL treatment, the expression levels of IL-6 and IL-1β were 20.68 and 2.69 times that of the control group, respectively (Figure 7A,B). For TNF-α and iNOS, 25 μg/mL was the optimal concentration, and the expression levels of TNF-αand iNOS were 2.36 and 3.16 times that of the control group, respectively (Figure 7C,D).

#### 2.6.3. Transcriptomic Analysis of the Antitumor Mechanisms of SPP-0.7A

**Figure 8:**
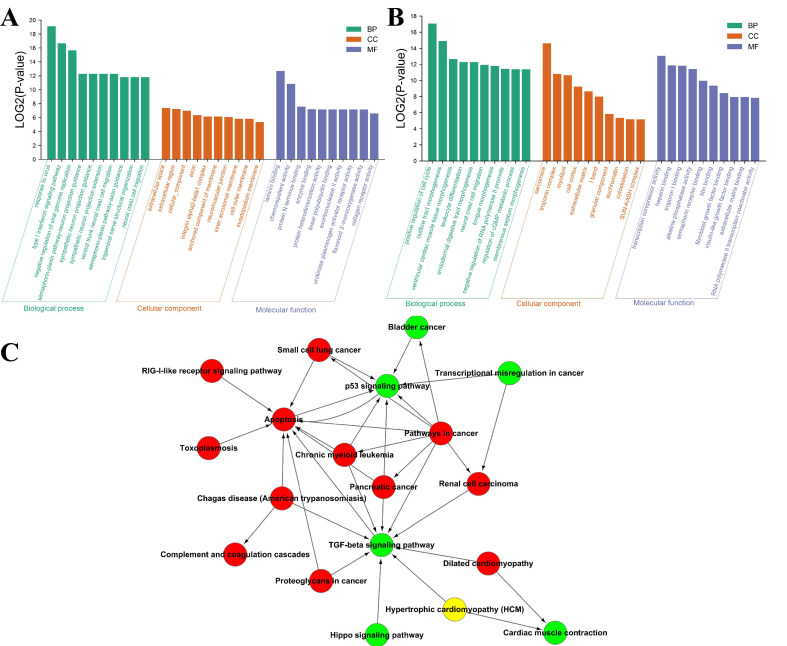
Enrichment analysis of DEGs in transcriptome. (**A**) GO enrichment of up-regulated DEGs in three major GO categories, including biological process (BP), molecular function (MF), and cell composition (CC). (**B**) GO enrichment of down-regulated DEGs. (**C**) Pathway network of KEGG enrichment of DEGs. The red circles represent the up-regulated pathways, the green circles represent the down-regulated pathways, and the yellow circle represents non-significantly difference pathway.

To screen the antitumor mechanisms and signal transduction pathways of SPPs on cancer cells, the transcriptome changes of A549 cells after adding SPP-0.7A were analyzed by RNA sequencing. 163 differentially expressed genes (DEGs) (FDR < 0.05, fold change > 1.5) between experimental group and control group were identified, including 81 up-regulated and 82 down-regulated DEGs. Gene Ontology (GO) enrichment analysis classified all DEGs. The top 10 significantly enriched GO terms in three major categories of biological process (BP), molecular function (MF), and cell composition (CC) were shown in Figure 8A,B. For up-regulated DEGs, genes related to “response to virus” (GO:0009615), “type I interferon signaling pathway” (GO:0060337), “negative regulation of viral genome replication” (GO:0045071) were the top 3 up-regulated GO terms, indicating that the cell stress response and cytokine secretion of cancer cells were activated by SPP-0.7A. For down-regulated DEGs, genes related to “positive regulation of cell cycle” (GO:0045787), “outflow tract morphogenesis” (GO:0003151), and “transcription corepressor activity” (GO:0003714) rank in the top three down-regulated GO terms, which indicated that the genes related to normal cell cycle and cell morphogenesis of cancer cells were inhibited. Meanwhile, some up-regulated GO terms were associated with “chemorepellent activity” (GO:0045499) and “semaphorin-plexin signaling pathway” (GO:0071526), and some of down-regulated GO terms were related to “troponin I binding” (GO:0031013), “alkaline phosphatase activity” (GO:0004035), “semaphorin receptor binding” (GO:0030215), “titin binding” (GO:0031432), “fibroblast growth factor binding” (GO:0017134), “insulin-like growth factor binding” (GO:0005520), which further indicated that SPP-0.7A might activate cellular stress response and cytokines secretion, and inhibit the cell cycle and morphogenesis of cancer cells.

These DEGs were also categorized into canonical pathways using Kyoto Encyclopedia of Genes and Genomes (KEGG) enrichment analysis. The significantly enriched pathways including 14 up-regulated pathway and 10 down-regulated pathway, as shown in Table 2 and Table 3. For up-regulated DEGs, pathways associated with cell apoptosis and defense, such as “apoptosis” (PATH:04210), “proteoglycans in cancer” (PATH:05205), “RIG-I-like receptor signaling pathway” (PATH:04622) were significantly activated. For down-regulated DEGs, pathways related to cancer transcriptional regulation and normal cell signaling pathways, such as “transcriptional misregulation in cancer” (PATH:05202), “hippo signaling pathway” (PATH:04390), “TGF-beta signaling pathway” (PATH:04350) were significantly suppressed. Meanwhile, some DEGs were found to be associated with some cancer or cancer pathway, like “pathways in cancer” (PATH:05200), “small cell lung cancer” (PATH:05222), “renal cell carcinoma” (PATH:05211), “pancreatic cancer” (PATH:05212), “bladder cancer” (PATH:05219), which further suggested that SPP-0.7A has antitumor activity. 

In addition, a pathway network was constructed to elucidate the interaction functional correlations of enrichment pathways (Figure 8C). The results showed that the up-regulated enrichment pathways related to apoptosis and cancer pathways were located on key nodes in the network, such as “apoptosis”, “pathways in cancer”, which has a key interaction relationship with down-regulated pathways. While in the down-regulated pathway, the “TGF-beta signaling pathway” and “p53 signaling pathway” (PATH:04115) were on the key nodes of the network.

## 3. Discussion

Numerous studies have focused on the evaluation of antitumor activity and further applications of polysaccharides from different sources [3,35,36]. Among them, seaweed polysaccharides are considered a new type of antitumor agent owing to their low toxicity and immunomodulatory activity, and have become a research hotspot [37,38]. Fan et al. found that *Sargassum fusiforme* polysaccharides (SFPs) have strong antitumor activity against nasopharyngeal carcinoma (NPC) and human hepatocellular carcinoma (HepG2). SFPs stimulate macrophages to secrete cytokines (IL-1 and TNF-a) and promote the proliferation of splenic lymphocytes [39,40]. Jiao et al. found that polysaccharides isolated from *Enteromorpha intestinalis* (EIPs) have significant antitumor effect on the growth of Sarcoma-180 solid tumors [41]. In the EIPs-treated mice, macrophage, and lymphocyte proliferation, and the production of TNF-α and NO were significantly enhanced [41]. These studies indicated that seaweed polysaccharides have both antitumor and immune-enhancing activities. However, most analyses of polysaccharide activities have focused only on a single activity (antitumor or immunomodulatory activity). The active components of polysaccharides that possess these two activities need to be further examined, and their antitumor mechanism needs to be further analyzed; these studies will contribute to their further application in the food industry and cancer therapy [42]. The results in this study showed that SPPs and SPP-0.7 have significant antitumor and immune-enhancing activities. For example, SPP-0.7 showed the highest growth-inhibitory effect against A549 and B16 cells. Moreover, SPP-0.7 exhibited the highest potency in stimulating macrophage proliferation, and the highest potency in stimulating lymphocyte proliferation. These data indicate that SPP-0.7 is the most active fraction of SPPs and might be suitable for use as a potential candidate natural agent for antitumor drug development.

As a traditional edible brown alga and herbal medicine, *S. pallidum* has been widely used as a dietary supplement in traditional Chinese medicine to treat tumors [18,19]. Moreover, SPPs have been shown to possess various beneficial effects, such as antioxidative [22,43], hypoglycemic [43,44], and antimicrobial activities [45]. However, only one study has focused on their antitumor properties. Ye et al. prepared SPPs by supercritical ultrasonic-aided extraction, CO_2_ extraction, and membrane separation technology, and found that the fractions SP-3-1 and SP-3-2 show higher antitumor activity than other fractions [20]. In addition, the immunomodulatory activity of SPPs is still unknown. The immunomodulatory activity of SPP on the growth of immune cells and serum cytokines remains unclear. This is not conducive to their further application, especially for the development of antitumor and immune-enhancing drugs in cancer therapy. In the present study, polysaccharide fractions from *S. pallidum* were isolated, and their antitumor and immune-enhancing properties were studied. The results show that SPPs have significant antitumor activity in all three types of cancer cells and induce cancer cell apoptosis. Additionally, the results suggested that SPPs enhance the activity of immune cells (macrophages and lymphocytes) and increase the levels of serum cytokines (IL-6, IL-1β, iNOS, and TNF-α). This is the first study on the antitumor and immune-enhancing activity of SPPs, which also contributes to the further development of this traditional Chinese herbal medicine and seaweed.

The antitumor effect of polysaccharides is a complex process, and various antitumor mechanisms correlated with each other [3]. Various antitumor mechanisms of polysaccharides have been reported previously [6,46,47]. Among them, enhancing host immune response and inducing tumor cell apoptosis has been considered as possible means of inhibiting tumor growth without harming the host [41]. Immunocytes, such as macrophages and lymphocytes, play important roles in inhibiting the proliferation of tumor cells in the body [48,49]. Cytokines play important roles in mediating the antitumor immune activity. For example, TNF-α can cause tumor-related endothelial cell apoptosis and tumor cell necrosis, and induce the expression of several immunomodulatory mediators [50,51]. iNOS plays a positive role in tumor apoptosis, and the up-regulation of iNOS was reported to stimulate apoptosis of osteosarcoma cells [52]. In this study, the fraction SPP-0.7 showed the strongest antitumor and immune-enhancing activities among the five polysaccharide fractions. It can significantly increase the proliferation rate of immune cells (macrophages and lymphocytes), increase the expression levels of serum cytokines (IL-6, IL-1β, iNOS, and TNF-α), and induce tumor cell apoptosis. Transcriptome sequencing analysis showed that genes and pathways related to cell apoptosis, cytokine secretion and cellular stress response of A549 cells were significantly up-regulated by SPP-0.7A treatment, indicating that SPP-0.7A induces apoptosis and defense processes. The genes and pathways related to cancer transcriptional regulation and normal cell signaling pathways were significantly supressed by polysaccharide, which indicated that polysaccharides can inhibit the normal physiological processes of tumor cells. In addition, genes related to RIG-I-like receptor signaling pathway, hippo signaling pathway, TGF-beta signaling pathway, and p53 signaling pathway were significantly expressed, which revealed that the antitumor activity of SPPs might be mediated via these pathways. 

The chemical composition and structural characteristics of polysaccharides have been confirmed to determine their biological activity [3,53]. Therefore, it is necessary to determine which group in the molecule plays an important role in conferring biological function [24]. As expected, significant changes in the ratio of total saccharides, the chemical composition of monosaccharides and the sulfated content were detected in all five polysaccharide fractions. The characteristics that make the SPP-0.7 activity superior to others may be due to its higher total sugar and sulfate content in saccharides. Sulfate was considered as the basis of biological activity in seaweed polysaccharides and oligosaccharides [54]. In addition, some typical polysaccharide signals, such as galactose signal, alkane signal, and pyran ring signal, may be one of the reasons for its excellent antitumor activity. It is worth noting that some of the chemical composition of SPP-0.7, such as the monosaccharides composition, are found to be different from the polysaccharides extracted from other *Sargassum* seaweed, such as *Sargassum thunbergii* [55] and *Sargassum stenophyllum* [56], which indicates the structure of SPP-0.7 may be new. However, this conclusion needs to be determined by a comprehensive structural analysis, such as 2D NMR. This is a shortcoming of our research. It is important to further determine the structural analysis for SPP-0.7 in the next experiments.

## 4. Materials and Methods

### 4.1. Preparation of Crude Polysaccharide

*S. pallidum* was collected from the coast of Qingdao (Shandong province, China). Species identification was performed by the laboratory assistant, Bing Li (College of Marine Science and Engineering, Qingdao Agricultural University, Qingdao, China). The collected samples were washed and dried at 60 °C in an oven. The dried algae were smashed and filtered through a 100-mesh sieve and processed using hot-water extraction methods [24]. Briefly, algal powder (200 g) was dipped into 20 volumes (*m*/*v*) of distilled water, and the solution was refluxed at approximately 90 °C for 5 h. The supernatant was concentrated under reduced pressure and dialyzed against distilled water for 72 h in dialysis membranes (molecular weight cut-off 3500). The dialyzed fraction was precipitated by the addition of four volumes of 95% (*v*/*v*) ethanol, washed twice with absolute ethanol, and then lyophilized to obtain the crude polysaccharides. 

### 4.2. Purification of Polysaccharides

The crude polysaccharides were deproteinated using the Sevag method [57]. The polysaccharides were then chromatographed on a DEAE-Sepharose fast-flow column (GE Healthcare, New York, NY, USA). The column was eluted using a step-wise gradient consisting of 0 M, 0.1 M, 0.3 M, 0.5 M, 0.7 M, 1 M, 1.3 M, 1.5 M, 1.7 M, and 2.0 M NaCl. The flow rate was 3.0 mL/min, and 10 mL fractions were collected. The polysaccharide fractions were identified using a phenol-sulfuric acid assay [58].

### 4.3. Chemical Composition Analysis

The levels of total carbohydrates were determined using the phenol-sulfuric acid method and d-glucose as standard [58]. The levels of sulfate were determined using the barium chloride-gelatin method [59].

The molar ratio of monosaccharides was determined by the 1-phenyl-3-methyl-5-pyrazolone (PMP) derivatization assay, as described previously, with slight modifications [60]. Briefly, 10 mg of polysaccharides was hydrolyzed in the presence of 4 mL of trifluoroacetic acid (TFA, 4 M) in an ampoule at 110 °C for 6 h. The hydrolyzed polysaccharides were dried and dissolved in 0.1 mL of distilled water. Then, the hydrolysate (100 μL) was derivatized by adding 100 μL of NaOH (0.3 M) and 120 μL of PMP methanol solution (0.5 M) and incubating at 70 °C for 1 h. The mixture was neutralized with 100 μL of HCl (0.3 M) and extracted with chloroform. Finally, the aqueous phase passed through a 0.45 μm membrane and the resulting solution (100 μL) was injected into a high-performance liquid chromatography (Shimadzu-20A, Shimadzu, Kyoto, Japan) equipped with a YMC-Pack ODS-AQ column (250 mm × 4.6 mm, 5 μm) (Agilent, Santa Clara, CA, USA). The mobile phase was composed of water and acetonitrile, and the flow rate was 1.0 mL/min. Monosaccharides (fucose, galacturonic acid, galactose, glucose, mannose, rhamnose, and xylose) (Sigma, St. Louis, MO, USA) were used as controls.

### 4.4. FT-IR Spectrometry and NMR Analysis

The polysaccharide samples were dried and ground in potassium bromide and pressed into pellets for spectral analysis [61]. FT-IR spectra were recorded using a Nicolet FT-IR 360 spectrophotometer (Thermo Fisher Scientific, Waltham, MA, USA). The scanning area was 400–4000 cm^−1^ (36 scans with a resolution of 6 cm^−1^).

The fraction SPP-0.7 was selected for further purification using Sephadex G-100 gel-filtration column (4 cm × 80 cm) (Solarbio, Beijing, China) according to its molecular distribution. Distilled water was used as the eluent at a flow rate of 0.5 mL/min. A phenol sulfuric acid assay was used to identify the purified fractions. Based on the chromatograph, the relevant fractions were pooled, dialyzed with water, and lyophilized.

The purified polysaccharides (50 mg) were dissolved in 0.5 mL of D_2_O for 3 h, and then ^1^H-NMR and ^13^C-NMR spectra were obtained at 500.13 MHz and 125.75 MHz on an NMR spectrometer (Avance NEO 500M, Bruker, Karlsruhe, Germany) at room temperature (26–28 °C).

### 4.5. Antitumor Activity Assay

#### 4.5.1. Cell Lines and Culture 

The human lung cancer cell line A549, human hepatoma cell line HepG2, and murine melanoma cell line B16 were provided by the Yellow Sea Fisheries Research Institute. The cell lines were maintained in RPMI-1640 medium containing 10% fetal bovine serum, 100 ng/mL streptomycin, and penicillin at 37 °C and 5% CO_2_.

#### 4.5.2. Cell Proliferation Assay

The antitumor activity of polysaccharide fractions on the three types of cancer cells was evaluated using the Cell Counting Kit-8 (CCK-8) (Saint-Bio, Shanghai, China) [62]. Briefly, 100 μL of cells was cultured in a 96-well plate at a concentration of 2 × 10^4^ cells/mL. After 24 h culture, different concentrations (25, 100, and 400 μg/mL) of the five polysaccharide fractions were slowly added to the cells and incubated for 24 h. Then, 20 μL of CCK-8 reagent was added to each well and incubation was continued for 4 h. After incubation, the absorbance was measured at 450 nm using a microplate reader (Thermo Fisher Scientific, Waltham, MA, USA). The antitumor activity defined as the rate inhibition of cell growth was calculated using the following formula:

Inhibition rate (%) = (1 − Ae/Ac) × 100 %,(1)

where Ae and Ac are the absorbances of the experimental and control groups, respectively.

#### 4.5.3. Apoptosis Assay

A549 cells were seeded in 6-well culture plates (1 × 10^5^ cells/mL) and treated with 0, 25, 100, and 400 μL/mL SPP-0.7A for 48 h. Cells were collected by centrifugation at 1700 rpm, washed three times with PBS, and stained with 5 μL of annexin V-FITC and 5 μL of propidium iodide for 15 min. Next, 400 μL of 1 × Annexin V binding buffer (TransGen Biotech, Beijing, China) was added and mixed, and the plates were placed in the dark. The apoptosis rate was analyzed within 1 h using a FACS Aria II flow cytometer (Becton Dickinson, Franklin Lakes, NJ, USA).

### 4.6. Immune-Enhancing Activity Assay 

#### 4.6.1. RAW264.7 Proliferation Assay

RAW 264.7 macrophages were provided by the Yellow Sea Fisheries Research Institute and cultured in DMEM medium in a cell incubator (37 °C, 5% CO_2_). 5 × 10^5^ cells/well were plated in 96-well plates and treated with 25, 100, and 400 μg/mL of each of the five fractions for 24 h. The control group was incubated with PBS instead of the polysaccharide solution. Twenty microliters of μL CCK-8 was added to each well and incubation continued for 4 h. Absorbance was measured at 450 nm using a microplate reader. The proliferation rate was calculated using the following formula:

Proliferation rate (%) = Ae/Ac × 100%,(2)

where Ae and Ac are the absorbances of the experimental and control groups, respectively.

#### 4.6.2. Mouse Lymphocyte Proliferation Assay

Four-week-old Kunming mice were purchased from the Qingdao Laboratory Animal Center (Qingdao, China). All mice were bred and housed under specific pathogen-free conditions at Qingdao Agricultural University, and all experiments were carried out in accordance with the protocols of the Institutional Animal Care and Use Committee of Qingdao Agricultural University (2018-192). Mice were anesthetized and euthanized by cervical dislocation, and the spleen was aseptically removed. Homogenic lymphocytes were obtained by mincing the spleens and filtering through a 200-mesh cell strainer.

In total, 2 × 10^6^ lymphocytes per well in 96-well plates were treated with 25, 100, and 400 μg/mL of each of the five fractions for 48 h. The control group was incubated with PBS instead of polysaccharides. Twenty microliters of μL CCK-8 was added to each well and incubation continued for 4 h. The absorbance was measured at 450 nm. 

#### 4.6.3. Assay of Cytokines Expression Level

The RT-PCR method was used to determine the cytokine levels secreted by RAW264.7 cells after incubation with SPP-0.7A. Briefly, cells were seeded in 6-well plates (3 × 10^5^ per well), and incubated with various concentrations of SPP-0.7A (25, 100, and 400 μg/mL) for 48 h. Cells in the control group were treated with PBS. Then, TRIzol was used to extract total RNA, which was transcribed into cDNA using a reverse transcription kit (Takara, Dalian, China) according to the manufacturer’s instructions. RT-PCR was performed with the incorporation of SYBR Green using Rotor-Gene Q (Qiagen, Duesseldorf, Germany). The expression levels of the cytokines IL-1β, IL-6, TNF-α, and iNOS were calculated using the comparative CT method [63]. β-Actin was used as the control gene. The primer sequences used are listed in Table 4.

### 4.7. Transcriptomic Assay and Analysis

Transcriptomic analysis of A549 cells between SPP-0.7A treatment group and control group were performed by the previously described methods [47]. Briefly, A549 cells were cultured at 2 × 10^5^ cell/well. The treated group of SPP-0.7A samples was added at a concentration of 100 μg/mL (the concentration with the best antitumor activity) for 48 h, and three independent biological replicates were performed. TRIzol was used to extract total RNA as described as part 4.6.3. The extracted RNA was purified and reverse transcribed before the analysis of transcriptome data [64]. The library construction and sequencing were carried out by Shanghai NovelBio Bio-Pharm Technology Co., Ltd (Shanghai, China). The transcriptomic library was paired-end sequenced in line based on the manufacturer’s instructions [64]. Reads that passed the quality control check were trimmed, filtered out, and mapped against the reference human genome as previously reported [65]. The DEG analysis was performed in EdgeR, with a FDR set at ≤0.05 and a threshold for a significant Fold Change (FC) set at ≥1.5 [66]. GO functional enrichment was conducted using Metascape (version 3.5, https://metascape.org/gp/index.html, accessed on 15 November 2021) [67] and KEGG pathway enrichment analysis was performed using KOBAS 3.0 (http://kobas.cbi.pku.edu.cn/kobas3/genelist/, accessed on 17 November 2021) [68]. Cytoscape (version 3.5.1, Cytoscape Consortium, CA, USA) was used to visualize the interaction functional correlations of enrichment pathways networks.

### 4.8. Statistical Analyses 

All experiments were repeated in triplicates and analyzed using SPSS 16.0 (SPSS Inc., Chicago, IL, USA). The results are shown as mean ± SD (standard deviation). Significant differences (*p* < 0.05) between the values were subjected to one-way analysis of variance and tested using Duncan’s test. The data (elution curve and biological tests) have passed the normality test and were determined to be non-normal in distribution (inhibitory rate, proliferation rate, and expression pattern), and then transformed into mean value.

## 5. Conclusions

In this study, polysaccharide fractions were isolated and purified from *S. pallidum*, a seaweed used in traditional Chinese herbal medicine. The physicochemical properties and antitumor and immune-enhancing activities were characterized. Among the various isolated polysaccharide fractions, SPP-0.7 showed the best antitumor and immune-enhancing activities and may be suitable as a potential candidate agent for cancer therapy; its anti-tumor mechanism was further studied. This research will enhance our understanding of the physicochemical properties and bioactivities of *S. pallidum* polysaccharides, which might be applied as potential bioactive components for antitumor drugs with immune-enhancing activity.

## Figures and Tables

**Table 1:** Yield and chemical composition of the five polysaccharide fractions extracted from *Sargassum pallidum*.

Sample	Yield (%)	Total Sugar (%)	Sulfate (%)	Monosaccharides Composition (Molar Ratio)						
				Man	Rha	GlcA	Glc	Gal	xyl	Fuc
SPP-0.3	11.90%	43.20%	5.39%	0.110	1	-	0.759	-	0.384	0.131
SPP-0.5	7.50%	67.16%	6.88%	0.902	0.512	-	0.699	0.733	0.273	1
SPP-0.7	5.90%	73.27%	9.57%	0.671	-	-	-	0.217	-	1
SPP-1	9.20%	62.59%	8.80%	-	1	-	0.012	0.002	0.003	-
SPP-2	2.30%	63.45%	13.79%	-	1	-	-	0.052	-	0.006

Man: mannose; Rha: rhamnose; Gal: galactose; Glc: glucose; Xyl: xylose; Fuc: fucose.

**Table 2:** KEGG enrichment of up-regulated DEGs show the significantly enriched pathways. The data are ranked according to the *p*-Value, which indicates the degree of enrichment.

PathwayID	PathwayTerm	*p*-Value	Enrichment
PATH:05222	Small cell lung cancer	0.0038138	10.869984
PATH:05200	Pathways in cancer	0.0064712	4.8860029
PATH:05145	Toxoplasmosis	0.0115104	7.1661376
PATH:04610	Complement and coagulation cascades	0.0225988	9.4845938
PATH:05211	Renal cell carcinoma	0.0238089	9.2136054
PATH:05212	Pancreatic cancer	0.0238089	9.2136054
PATH:04622	RIG-I-like receptor signaling pathway	0.0244236	9.0838364
PATH:05220	Chronic myeloid leukemia	0.0256719	8.8349641
PATH:00980	Metabolism of xenobiotics by cytochrome P450	0.0315934	7.8652729
PATH:05410	Hypertrophic cardiomyopathy (HCM)	0.032281	7.7705106
PATH:05414	Dilated cardiomyopathy	0.037253	7.1661376
PATH:04210	Apoptosis	0.037253	7.1661376
PATH:05205	Proteoglycans in cancer	0.0413872	4.2996825
PATH:05142	Chagas disease (American trypanosomiasis)	0.0471805	6.2616736

**Table 3:** KEGG enrichment of down-regulated DEGs show the significantly enriched pathways. The data are ranked according to the *p*-Value, which indicates the degree of enrichment.

PathwayID	PathwayTerm	*p*-Value	Enrichment
PATH:00790	Folate biosynthesis	0.0015041	42.0621118
PATH:04390	Hippo signaling pathway	0.0029298	7.697641375
PATH:04350	TGF-beta signaling pathway	0.0043803	10.27098079
PATH:05219	Bladder cancer	0.0110488	14.02070393
PATH:04115	p53 signaling pathway	0.0264354	8.659846547
PATH:05202	Transcriptional misregulation in cancer	0.0296241	4.907246377
PATH:04260	Cardiac muscle contraction	0.0337453	7.54960981
PATH:05410	Hypertrophic cardiomyopathy (HCM)	0.0376566	7.094814039
PATH:05414	Dilated cardiomyopathy	0.0434007	6.542995169
PATH:04015	Rap1 signaling pathway	0.0445293	4.146968769

**Table 4:** List of primers. F represents forward primer and R represents reverse primer.

Gene	Primer	Sequence (5′–3′)
IL-6	F	CATGTTCTCTGGGAAATCGTGG
	R	AACGCAACTAGGTTTGCCGAGTA
IL-1β	F	GGGATGATGATGATAACCTG
	R	TTGTCGTTGCTTGGTTCTCCT
TNF-α	F	GATCTCAAAGCAAACCAACTAGTG
	R	CTCCAGCTGGAAGACTCCCAG
iNOS	F	GGTCTTCCTGGGCTCGATCTG
	R	GCCGTGGCCAACATGCTACT
β-actin	F	GCAGAAGGAGATCACTGCCCT
	R	GCTGATCCACATCTGCTGGAA

## Data Availability

Not applicable.

## Sample Availability Statement

Samples of the compounds are available from the authors.

## References

[1] Bray F., Ferlay J., Soerjomataram I., Siegel R.L., Torre L.A., Jemal A. (2018). Global cancer statistics 2018: GLOBOCAN estimates of incidence and mortality worldwide for 36 cancers in 185 countries. CA-Cancer J. Clin..

[2] Jemal A., Bray F., Center M.M., Ferlay J., Ward E., Forman D. (2011). Global cancer statistics. CA: A Cancer J. Clin..

[3] Li N., Wang C., Georgiev M.I., Bajpai V.K., Tundis R., Simal-Gandara J., Lu X., Xiao J., Tang X., Qiao X. (2021). Advances in dietary polysaccharides as anticancer agents: Structure-activity relationship. Trends Food Sci. Technol..

[4] Ikeguchi M., Yamamoto M., Arai Y., Maeta Y., Ashida K., Katano K., Miki Y., Kimura T. (2011). Fucoidan reduces the toxicities of chemotherapy for patients with unresectable advanced or recurrent colorectal cancer. Oncol. Lett..

[5] Bagchi D., Bagchi M., Stohs S.J., Das D.K., Ray S.D., Kuszynski C.A., Joshi S.S., Pruess H.G. (2000). Free radicals and grape seed proanthocyanidin extract: Importance in human health and disease prevention. Toxicology.

[6] Zong S., Li J., Ye Z., Zhang X., Yang L., Chen X., Ye M. (2020). Lachnum polysaccharide suppresses S180 sarcoma by boosting anti-tumor immune responses and skewing tumor-associated macrophages toward M1 phenotype. Int. J. Biol. Macromol..

[7] Starke A., Lindenmeyer M.T., Segerer S., Neusser M.A., Rüsi B., Schmid D.M., Cohen C.D., Wüthrich R.P., Fehr T., Waeckerle-Men Y. (2010). Renal tubular PD-L1 (CD274) suppresses alloreactive human T-cell responses. Kidney Int..

[8] Gonzalez-Gugel E., Saxena M., Bhardwaj N. (2016). Modulation of innate immunity in the tumor microenvironment. Cancer Immunol. Immunother..

[9] Liu C., Cui Y., Pi F., Cheng Y., Guo Y., Qian H. (2019). Extraction, Purification, Structural Characteristics, Biological Activities and Pharmacological Applications of Acemannan, a Polysaccharide from Aloe vera: A Review. Molecules.

[10] Rong Y., Yang R., Yang Y., Wen Y., Liu S., Li C., Hu Z., Cheng X., Li W. (2019). Structural characterization of an active polysaccharide of longan and evaluation of immunological activity. Carbohydr. Polym..

[11] Xie J.-H., Jin M.-L., Morris G.A., Zha X.-Q., Chen H.-Q., Yi Y., Li J.-E., Wang Z.-J., Gao J., Nie S.-P. (2016). Advances on Bioactive Polysaccharides from Medicinal Plants. Crit. Rev. Food Sci. Nutr..

[12] Janeway C.A., Medzhitov R. (2002). Innate immune recognition. Annu. Rev. Immunol..

[13] Zhang J., Song Z., Li Y., Zhang S., Bao J., Wang H., Dong C., Ohizumi Y., Xu J., Guo Y. (2021). Structural analysis and biological effects of a neutral polysaccharide from the fruits of Rosa laevigata. Carbohydr. Polym..

[14] Kim J.Y., Yoon Y.D., Ahn J.M., Kang J.S., Park S.-K., Lee K., Song K.B., Kim H.M., Han S.-B. (2007). Angelan isolated from Angelica gigas Nakai induces dendritic cell maturation through toll-like receptor 4. Int. Immunopharmacol..

[15] Li W.-J., Chen Y., Nie S.-P., Xie M.-Y., He M., Zhang S.-S., Zhu K.-X. (2011). Ganoderma atrum polysaccharide induces anti-tumor activity via the mitochondrial apoptotic pathway related to activation of host immune response. J. Cell. Biochem..

[16] Yuan D., Li C., Huang Q., Fu X. (2020). Ultrasonic degradation effects on the physicochemical, rheological and antioxidant properties of polysaccharide from Sargassum pallidum. Carbohydr. Polym..

[17] Luo D., Yuan X., Zeng Y., Nie K., Li Z., Wang Z. (2016). Structure elucidation of a major fucopyranose-rich heteropolysaccharide (STP-II) from Sargassum thunbergii. Carbohydr. Polym..

[18] Cao Y., Duan J.A., Fan X.S., Guo J.M., Shu-Lan S.U. (2014). Exploration and Analysis of the Herbal Nature and Application Characteristics of Sargassum. Chin. J. Exp. Tradit. Med. Formulae.

[19] Ma Y., Zhang Y., Zhai Y., Zhu Z., Pan Y., Qian D., Su S., Fan X., Duan J. (2016). Development of a UPLC-TQ/MS Approach for the Determination of Eleven Bioactive Components in Haizao Yuhu Decoction Plus-Minus Haizao and Gancao Drug Combination after Oral Administration in a Rat Model of Hypothyroidism. Molecules.

[20] Ye H., Wang K., Zhou C., Liu J., Zeng X. (2008). Purification, antitumor and antioxidant activities in vitro of polysaccharides from the brown seaweed Sargassum pallidum. Food Chem..

[21] Li C., Li X., You L., Fu X., Liu R.H. (2017). Fractionation, preliminary structural characterization and bioactivities of polysaccharides from Sargassum pallidum. Carbohydr. Polym..

[22] Cao C., Li C., Chen Q., Huang Q., Pérez M.E.M., Fu X. (2019). Physicochemical characterization, potential antioxidant and hypoglycemic activity of polysaccharide from Sargassum pallidum. Int. J. Biol. Macromol..

[23] Xiao H., Fu X., Cao C., Li C., Chen C., Huang Q. (2019). Sulfated modification, characterization, antioxidant and hypoglycemic activities of polysaccharides from Sargassum pallidum. Int. J. Biol. Macromol..

[24] Song L., Chen X., Liu X., Zhang F., Hu L., Yue Y., Li K., Li P. (2015). Characterization and Comparison of the Structural Features, Immune-Modulatory and Anti-Avian Influenza Virus Activities Conferred by Three Algal Sulfated Polysaccharides. Mar. Drugs.

[25] Seedevi P., Moovendhan M., Sudharsan S., Sivasankar P., Sivakumar L., Vairamani S., Shanmugam A. (2018). Isolation and chemical characteristics of rhamnose enriched polysaccharide from Grateloupia lithophila. Carbohydr. Polym..

[26] Wang J., Li Q., Bao A., Liu X., Zeng J., Yang X., Yao J., Zhang J., Lei Z. (2016). Synthesis of selenium-containing Artemisia sphaerocephala polysaccharides: Solution conformation and anti-tumor activities in vitro. Carbohydr. Polym..

[27] Rochas C., Lahaye M., Yaphe W. (1986). Sulfate Content of Carrageenan and Agar Determined by Infrared Spectroscopy. Bot. Mar..

[28] Ren Y.-Y., Zhu Z.-Y., Sun H.-Q., Chen L.-J. (2017). Structural characterization and inhibition on α-glucosidase activity of acidic polysaccharide from Annona squamosa. Carbohydr. Polym..

[29] Feng Z.X., Yang J.F., Ni L.M., Gao Q., Liu Z.J. (2021). The chemical and structural transformation of bamboo wastes during torrefaction process. Environ. Prog. Sustain. Energy.

[30] Kuang H., Jiao Y., Wang W., Wang F., Chen Q. (2020). Characterization and antioxidant activities of intracellular polysaccharides from Agaricus bitorquis (QuéL.) Sacc. Chaidam ZJU-CDMA-12. Int. J. Biol. Macromol..

[31] El Rashed Z., Lupidi G., Kanaan H., Grasselli E., Canesi L., Khalifeh H., Demori I. (2021). Antioxidant and Antisteatotic Activities of a New Fucoidan Extracted from Ferula hermonis Roots Harvested on Lebanese Mountains. Molecules.

[32] Hong Y., Ying T. (2019). Isolation, molecular characterization and antioxidant activity of a water-soluble polysaccharide extracted from the fruiting body of Termitornyces albuminosus (Berk.) Heim. Int. J. Biol. Macromol..

[33] Ahrazem O., Prieto A., Leal J.A., Jiménez-Barbero J., Bernabé M. (2002). Fungal cell wall galactomannan isolated from Apodus deciduus. Carbohydr. Res..

[34] Zych K., Toukach F.V., Arbatsky N.P., Kolodziejska K., Senchenkova S.N., Shashkov A.S., Knirel Y.A., Sidorczyk Z. (2001). Structure of the O-specific polysaccharide of Proteus mirabilis D52 and typing of this strain to Proteus serogroup O33. Eur J. Biochem.

[35] Zhang S., Zhang H., Shi L., Li Y., Tuerhong M., Abudukeremu M., Cui J., Li Y., Jin D.-Q., Xu J. (2021). Structure features, selenylation modification, and improved anti-tumor activity of a polysaccharide from Eriobotrya japonica. Carbohydr. Polym..

[36] Liu X.-C., Zhu Z.-Y., Liu Y.-L., Sun H.-Q. (2019). Comparisons of the anti-tumor activity of polysaccharides from fermented mycelia and cultivated fruiting bodies of Cordyceps militaris in vitro. Int. J. Biol. Macromol..

[37] Saeed M., Arain M.A., Ali Fazlani S., Marghazani I.B., Umar M., Soomro J., Bhutto Z.A., Soomro F., Noreldin A.E., Abd El-Hack M.E. (2021). A comprehensive review on the health benefits and nutritional significance of fucoidan polysaccharide derived from brown seaweeds in human, animals and aquatic organisms. Aquac. Nutr..

[38] Jin J.-O., Chauhan P.S., Arukha A.P., Chavda V., Dubey A., Yadav D. (2021). The Therapeutic Potential of the Anticancer Activity of Fucoidan: Current Advances and Hurdles. Mar. Drugs.

[39] Fan S., Yu G., Nie W., Jin J., Chen L., Chen X. (2018). Antitumor activity and underlying mechanism of Sargassum fusiforme polysaccharides in CNE-bearing mice. Int. J. Biol. Macromol..

[40] Fan S., Zhang J., Nie W., Zhou W., Jin L., Chen X., Lu J. (2017). Antitumor effects of polysaccharide from Sargassum fusiforme against human hepatocellular carcinoma HepG2 cells. Food Chem. Toxicol..

[41] Jiao L., Li X., Li T., Jiang P., Zhang L., Wu M., Zhang L. (2009). Characterization and anti-tumor activity of alkali-extracted polysaccharide from Enteromorpha intestinalis. Int. Immunopharmacol..

[42] Li S., Xiong Q., Lai X., Li X., Wan M., Zhang J., Yan Y., Cao M., Lu L., Guan J. (2016). Molecular Modification of Polysaccharides and Resulting Bioactivities. Compr. Rev. Food Sci. Food Saf..

[43] Xiao H., Chen C., Li C., Huang Q., Fu X. (2019). Physicochemical characterization, antioxidant and hypoglycemic activities of selenized polysaccharides from Sargassum pallidum. Int. J. Biol. Macromol..

[44] Cao C., Huang Q., Zhang B., Li C., Fu X. (2018). Physicochemical characterization and in vitro hypoglycemic activities of polysaccharides from Sargassum pallidum by microwave-assisted aqueous two-phase extraction. Int. J. Biol. Macromol..

[45] Zhang K., Yuan D., Li C., Fu X. (2021). Physicochemical properties and bioactivity of polysaccharides from Sargassum pallidum by fractional ethanol precipitation. Int. J. Food Sci. Technol..

[46] Senthilkumar K., Manivasagan P., Venkatesan J., Kim S.-K. (2013). Brown seaweed fucoidan: Biological activity and apoptosis, growth signaling mechanism in cancer. Int. J. Biol. Macromol..

[47] Chen R., Wu J., Lu C., Yan T., Qian Y., Shen H., Zhao Y., Wang J., Kong P., Zhang X. (2021). Systematic Transcriptome Analysis Reveals the Inhibitory Function of Cinnamaldehyde in Non-Small Cell Lung Cancer. Front. Pharmacol..

[48] Shu G., Jiang S., Mu J., Yu H., Duan H., Deng X. (2018). Antitumor immunostimulatory activity of polysaccharides from Panax japonicus C. A. Mey: Roles of their effects on CD4+ T cells and tumor associated macrophages. Int. J. Biol. Macromol..

[49] Schepetkin I.A., Quinn M.T. (2006). Botanical polysaccharides: Macrophage immunomodulation and therapeutic potential. Int. Immunopharmacol..

[50] Baugh J., Bucala R. (2001). Mechanisms for modulating TNF alpha in immune and inflammatory disease. Curr. Opin. Drug Discov. Dev..

[51] Lejeune F.J., Liénard D., Matter M., Rüegg C. (2006). Efficiency of recombinant human TNF in human cancer therapy. Cancer Immun..

[52] Chu W., Cao L., Daokun G., Zhao J. (2021). iNOS Promotes the Development of Osteosarcoma via Wntβ-Catenin Pathway. J. Immunol. Res..

[53] Tabarsa M., Han J.H., Kim C.Y., You S.G. (2012). Molecular characteristics and immunomodulatory activities of water-soluble sulfated polysaccharides from Ulva pertusa. J. Med. Food.

[54] Percival E. (1979). The polysaccharides of green, red and brown seaweeds: Their basic structure, biosynthesis and function. Br. Phycol. J..

[55] Jin W., Liu B., Li S., Chen J., Tang H., Jiang D., Zhang Q., Zhong W. (2018). The structural features of the sulfated heteropolysaccharide (ST-1) from Sargassum thunbergii and its neuroprotective activities. Int. J. Biol. Macromol..

[56] Duarte M.E.R., Cardoso M.A., Noseda M.D., Cerezo A.S. (2001). Structural studies on fucoidans from the brown seaweed Sargassum stenophyllum. Carbohydr. Res..

[57] Alam N., Gupta P.C. (1986). Structure of a water-soluble polysaccharide from the seeds of Cassia angustifolia. Planta Med..

[58] Dubois M., Gilles H.A., Hamilton J.K., Rebers P.A., Smith F. (1956). Colorimetric Method for Determination of Sugars and Related Substances. Anal. Chem..

[59] Yan W., Niu Y., Lv J., Xie Z., Jin L., Yao W., Gao X., Yu L. (2013). Characterization of a heteropolysaccharide isolated from diploid Gynostemma pentaphyllum Makino. Carbohydr. Polym..

[60] Zhang W., Oda T., Yu Q., Jin J.O. (2015). Fucoidan from Macrocystis pyrifera has powerful immune-modulatory effects compared to three other fucoidans. Mar. Drugs.

[61] Chen Y., Xiong W., Zeng L., Wang D., Liu J., Wu Y., Hu Y. (2014). Comparison of Bush Sophora Root polysaccharide and its sulfate’s anti-duck hepatitis A virus activity and mechanism. Carbohydr. Polym..

[62] Gao X., Qi J., Ho C.-T., Li B., Mu J., Zhang Y., Hu H., Mo W., Chen Z., Xie Y. (2020). Structural characterization and immunomodulatory activity of a water-soluble polysaccharide from Ganoderma leucocontextum fruiting bodies. Carbohydr. Polym..

[63] Livak K.J., Schmittgen T.D. (2001). Analysis of relative gene expression data using real-time quantitative PCR and the 2(-Delta Delta C(T)) Method. Methods.

[64] Gao Y., Xu H., Shen Y., Wang J. (2013). Transcriptomic analysis of rice (Oryza sativa) endosperm using the RNA-Seq technique. Plant. Mol. Biol..

[65] Pauletto M., Tolosi R., Giantin M., Guerra G., Barbarossa A., Zaghini A., Dacasto M. (2020). Insights into Aflatoxin B1 Toxicity in Cattle: An In Vitro Whole-Transcriptomic Approach. Toxins.

[66] Robinson M.D., McCarthy D.J., Smyth G.K. (2010). edgeR: A Bioconductor package for differential expression analysis of digital gene expression data. Bioinformatics.

[67] Tripathi S., Pohl M.O., Zhou Y., Rodriguez-Frandsen A., Wang G., Stein D.A., Moulton H.M., DeJesus P., Che J., Mulder L.C.F. (2015). Meta- and Orthogonal Integration of Influenza “OMICs” Data Defines a Role for UBR4 in Virus Budding. Cell Host Microbe.

[68] Xie C., Mao X., Huang J., Ding Y., Wu J., Dong S., Kong L., Gao G., Li C.-Y., Wei L. (2011). KOBAS 2.0: A web server for annotation and identification of enriched pathways and diseases. Nucleic Acids Res..

